# Can a Symbiont (Also) Be Food?

**DOI:** 10.3389/fmicb.2019.02539

**Published:** 2019-11-07

**Authors:** Kim L. Hoang, Levi T. Morran, Nicole M. Gerardo

**Affiliations:** Department of Biology, O. Wayne Rollins Research Center, Emory University, Atlanta, GA, United States

**Keywords:** symbiont ingestion, beneficial microbes, food, symbiont farming, symbiont acquisition

Beneficial symbionts exist in many different forms, ranging from vertically-transmitted intracellular bacteria to environmentally-grown fungi. In many symbioses, the host ingests its symbiont. Symbiont ingestion appears to present a dilemma: symbionts may no longer gain from associations when acting as a food source, perhaps rendering the interaction unstable or disqualifying the interaction as a symbiosis altogether. So, can a symbiont serve as a food source and still be a symbiont? Contrary to perception, we argue that ingestion does not preclude the evolution of beneficial interactions beyond simply host nutrition.

## What Is a Microbial Symbiosis?

Symbiosis is the long-term association between two organisms over evolutionary time. Generally, in a microbial symbiosis, the microbial partner (symbiont) is in a relationship with a larger organism, the host. Most hosts typically interact with a large population of clonal symbionts. These symbioses exist on a spectrum, from mutualism to parasitism, and placement along that spectrum often depends on the ecological context. Symbionts that associate with hosts are typically bacteria and fungi, although viruses can also be beneficial (Oliver et al., 2009; Bondy-Denomy and Davidson, 2014).

## Do Symbionts Have to Benefit in a Symbiosis?

A mutualism indicates that an interaction results in a net positive fitness outcome for both partners. However, not all symbioses are mutualistic. Beneficial symbionts do not have to gain fitness for the association to exist (Mushegian and Ebert, 2016). For example, exposure to gut microbiota may help the host immune system respond properly (Kanther et al., 2011; Yilmaz et al., 2014), but the microbes may not benefit more from being host-associated compared to growing outside the host. Furthermore, some symbionts are exploited by their hosts, such that the symbiont exhibits a fitness cost when in symbiosis compared to when free-living. The algal endosymbiont *Chlorella*, for example, produces metabolites from photosynthesis that are utilized by its protist host, *Paramecium bursaria*, in exchange for nitrogen compounds. Despite this metabolic exchange, at high light intensity, *Chlorella* grows to higher levels in the host's absence than in its presence (Lowe et al., 2016). The idea that symbionts have to gain from the association may have been exacerbated by the common use of “mutualism” to describe symbioses, despite a lack of empirical evidence in most systems for increased symbiont fitness when interacting with the host (Garcia and Gerardo, 2014).

## Where Do Symbionts Reside?

Beneficial symbionts help their hosts by making the environment more habitable for the host, such as through nutrient provisioning or protection from enemies. Many symbionts reside inside the host, such as in the gut, or within specialized cells that hosts have evolved to accommodate symbionts. However, symbionts can also dwell their host's surface or proliferate in the external environment. These external symbionts can still be dependent on the host for growth and survival.

## Where Do Symbionts Come From?

Symbiont populations can spread between hosts vertically, horizontally, or through mixed-mode transmission (Ebert, 2013). Vertical transmission is direct transmission of symbiont from parent to offspring. Hosts obtain symbionts horizontally from other, non-parental hosts or from the environment. While horizontally transmitted symbionts are passaged extracellularly, vertical transmission does not always require the symbiont to be inherited intracellularly. For example, a bacterial symbiont can be transmitted vertically by an insect mother, such as in firebugs, when she smears the bacteria onto her eggs' surfaces, which are then ingested by her offspring when they hatch (Salem et al., 2015).

## Can Symbionts be Ingested? Why Is This Important to Consider?

Food is any substance that provides nutrients to the organism ingesting it. Here, we use “ingestion” to mean the intake of a substance, and “digestion” to mean the catabolism of the substance into materials that can be used. Symbiont ingestion appears counterintuitive: it implies subsequent digestion of the symbiont, thereby preventing further interactions, or symbiosis, with the host. In many symbioses, the symbiont does directly provide nutrients to its host through digestion (e.g., fungus-growing ants, see below). However, in other symbioses, the symbiont is acquired through ingestion but provides benefits other than, or in addition to, serving as a food source. For example, Sirex woodwasps deposit their fungal symbiont into decaying trees and ingest both the wood and the symbiont to obtain digestive enzymes from the fungi that help them breakdown plant compounds (Kukor and Martin, 1983). While these symbioses deviate from the more commonly known vertically-transmitted, intracellular symbioses, the hosts and symbionts can establish stable, long-term beneficial associations with one another. Below, we outline cases where the symbiont is ingested, either as a food, or as a route of acquisition by the host, and demonstrate that there may be a fine line between symbiont and food in many associations.

## Farming: Where the Symbiont Is the Food

### Could a Host's Primary Food Source Be Considered a Symbiont?

In farming symbioses, the host organism farms its microbial partner, such that the propagation of the microbe supplies the host with a renewable food source or other resources. When microbes are farmed, the microbial partner is commonly referred to as the symbiont (Mueller et al., 2004; Nobre et al., 2011; Qiu et al., 2016). Here, we define farming as growing and active tending (e.g., crop propagation, weeding, fertilizing) of symbionts for resources. Under this definition, many examples of domesticators and crops can be viewed as symbioses. However, an important point is that for the association to be considered a symbiosis, the symbiont should be a crucial determinant of host fitness at least under some environmental conditions. The symbiont can also be dependent on the host for growth and reproduction, resulting in a two-way obligate symbiosis. Important from an evolutionary perspective, the symbiont population is often clonal or lacking in genetic variation, so even if portions of the population are digested, the remaining cells are still able to reproduce and effectively maintain fitness.

Insects are some of the most well-studied groups of organisms that farm their symbionts, particularly fungi. For example, fungus-growing ants have an obligate association with their cultivated fungi—these fungi, the “cultivars,” are tended by the ants, where they supplement the cultivars with beneficial substrates, minimize competition with other fungi, and combat pathogens of the cultivar (Caldera et al., 2009). The ants in turn feed on the cultivars. Neither partner can exist without the other (Weber, 1966). This dependency is further reinforced by genomic alterations in the ants: the ants no longer have certain nutrient acquisition genes and must depend on the fungus (Suen et al., 2011). Because the fungus is clonally propagated, it does not lose fitness as the ants only feed on some of the cultivar, and undigested cultivar will be vertically transmitted to the next generation. As a result, despite serving primarily as food, the host ant and cultivar fungi have been coevolving with each other for millions of years (Schultz and Brady, 2008). Similarly, termites, ambrosia beetles, and Brazilian stingless bees have been shown to farm fungi (Aanen et al., 2002; Six, 2012; Menezes et al., 2015).

### Are There Other Farming Symbioses Besides Fungus-Farming Insects?

Examples in other systems include primitive forms of farming, where there is no evidence for active tending of the food source, but growth of the particular resource (symbiont) in the presence of the host is identified. For example, the social amoeba, *Dictyostelium discoideum*, farms its bacterial food source through dispersion, seeding, and careful harvesting (Brock et al., 2011, 2013; Stallforth et al., 2013). Marsh snails promote growth of consumable fungi by preparing fungal growing substrates and supplementing them with fecal pellets (Silliman and Newell, 2003). Lastly, the mitochondrion is proposed to have evolved from the symbiosis of an archaeon host and alphaproteobacteria prey. Theoretical models predict that the ancient association began from phagocytosis of the bacteria and their subsequent farming within the archaeon (Zachar et al., 2018). Therefore, interactions that begin as predation can evolve into a long-term symbiosis.

## Ingestion as a Route of Acquisition: Where the Benefit Is not Reliant on Symbiont Digestion

### Can Ingested Symbionts Benefit Their Hosts When Not Digested?

An intimate association between a host and its symbiont requires close physical proximity. Ingestion of symbionts facilitates this association by bringing the symbiont directly into the host. Many ingested symbionts provide their hosts with benefits that are not directly related to symbiont digestion. For example, some insect species produce bacteria-containing capsules, from which bacteria are ingested by the offspring when they hatch (Salem et al., 2015). Subsequently, the bacteria colonize a specific section of the gut and aid in the host's development (Fukatsu and Hosokawa, 2002). Several marine organisms, such as corals, gastropods, anemones, and jellyfish, also obtain their photosynthetic symbionts through ingestion (Colley and Trench, 1985; Abrego et al., 2009; Banaszak et al., 2013; Hambleton et al., 2014).

### Can a Symbiont Be Food While Also Providing Other Benefits?

Even ingested symbionts that benefit the host through means other than nutrition can be digested. For example, *Steinernema* spp. nematodes form a mutualism with the bacterium *Xenorhabdus nematophila*, which helps the nematode infect and kill insects by suppressing insect immunity, benefiting both the nematode and the bacterium. The nematode picks up *X. nematophila* through ingestion, and may obtain nutrients directly from bacterial digestion or from the breakdown of the insect cadaver by bacterial enzymes (Forst et al., 1997; Hussa and Goodrich-Blair, 2013). Ingestion ultimately serves as a method in which hosts can obtain symbionts and gain additional functions through them. Table 1 provides examples where symbionts can become food while helping their hosts through other means. Additionally, it is unclear whether portions of the gut microbiota [e.g., of humans, ruminants, coprophagic animals (Lozupone et al., 2012; Onchuru et al., 2018; Clemmons et al., 2019)] are digested.

**Table 1:** Examples of symbioses where the symbiont serves as food in addition to non-food functions.

**Host**	**Symbiont**	**Symbiont non-food function**	**References**
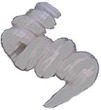			
*Paracatenula* flatworm	*Ca*. Riegeria santandreae bacterium	Provisioning of carbon and sugars	Jäckle et al., 2019
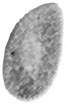			
*Paramecium bursaria* protist	*Chlorella* algae	Provisioning of metabolites from photosynthesis	Kodama and Fujishima, 2008
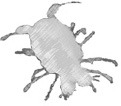			
*Santia* spp. isopod crustacean	*Cyanobacteria*	Production of chemical compounds that repel fish predators of the host	Lindquist et al., 2005
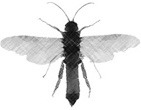			
Sirex woodwasps	Fungal symbiont	Digestive enzymes that breakdown plant compounds from host diet	Kukor and Martin, 1983
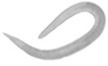			
*Steinernema* spp. nematodes	*Xenorhabdus nematophila* bacterium	Aid host in infecting, killing, and digesting insects	Forst et al., 1997

### Can a Long-Term Association Persist if the Majority of the Symbiont Population Does Not Remain in the Host Throughout the Host's Lifespan?

Symbionts obtained from ingestion may vary in the amount of time they remain in a host. While some hosts have physical structures that house symbionts, others allow symbionts to proliferate throughout the gut. In either case, individual symbionts have the potential to exit or be digested. While ingestion may lead to a relatively brief interaction, a long-term association across generations is still possible even when most of the symbiont population is transient. For example, the bobtail squid-*Vibrio fischeri* association is considered a canonical symbiosis despite a high rate of symbiont turnover within the host. A newborn squid picks up the bacteria from the environment each night, and, similar to symbioses where the symbiont is ingested [e.g., *Steinernema* spp. nematodes (Martens et al., 2003)], few bacterial cells enter the host (Wollenberg and Ruby, 2009). The bacteria then colonize the host's light organ, but over 95% are subsequently expelled the next morning (Lee and Ruby, 1994). Indeed, a symbiosis can form and persist for all or a portion of a single host's life span. Because microbes typically have shorter generation times than their hosts, occupying the host for a brief period of time can still result in multiple microbial generations within hosts.

## Conclusion

Here, we have presented examples of symbioses where the host ingests its symbiont either to obtain nutrients, live microbes, or both. Ingestion does not prevent the symbiont from coevolving with its host across evolutionary time. In the case of farming symbioses, the symbiont is propagated outside of the host and has the potential to evolve in response to its host. When the symbiont is ingested as a means of acquisition, the symbiont can be compartmentalized to specific locations within the host or colonize the host gut, where it also maintains the potential to respond to selective pressures from the host. Because symbiont populations tend to be clonal, digestion of some individuals does not limit the benefits the symbiont may receive, or symbiosis altogether. This is particularly important for associations where the symbiont serves as food in addition to other roles, where the host does not digest all of the symbiont population at once. Ultimately, ingestion is one of the many ways in which hosts and symbionts interact with one another, which can lead to intimate associations similar to intracellular symbioses.

## Author Contributions

KH and LM conceived the idea for the article. KH wrote the manuscript with input from LM and NG.

### Conflict of Interest

The authors declare that the research was conducted in the absence of any commercial or financial relationships that could be construed as a potential conflict of interest.
